# Dose reduction for CT coronary calcium scoring with a calcium-aware image reconstruction technique: a phantom study

**DOI:** 10.1007/s00330-020-06709-9

**Published:** 2020-02-19

**Authors:** Ronald Booij, Niels R. van der Werf, Ricardo P. J. Budde, Daniel Bos, Marcel van Straten

**Affiliations:** 1 Department of Radiology & Nuclear Medicine, Erasmus MC, P.O. Box 2240, 3000 CA Rotterdam, The Netherlands; 2 Department of Radiology, University Medical Center Utrecht, Utrecht, The Netherlands; 3 Department of Epidemiology, Erasmus MC, Rotterdam, The Netherlands

**Keywords:** Computed x-ray tomography, Coronary artery disease, Radiation dosage, Diagnostic imaging

## Abstract

**Objective:**

To assess the dose reduction potential of a calcium-aware reconstruction technique, which aims at tube voltage-independent computed tomography (CT) numbers for calcium.

**Methods and materials:**

A cardiothoracic phantom, mimicking three different patient sizes, was scanned with two calcium inserts (named D100 and CCI), containing calcifications varying in size and density. Tube voltage was varied both manually (range 70–150 and Sn100 kVp) and automatically. Tube current was automatically adapted to maintain reference image quality defined at 120 kVp. Data was reconstructed with the standard reconstruction technique (kernel Qr36) and the calcium-aware reconstruction technique (kernel Sa36). We assessed the radiation dose reduction potential (volumetric CT dose index values (CTDIvol)), noise (standard deviation (SD)), mean CT number (HU) of each calcification, and Agatston scores for varying kVp. Results were compared with the reference acquired at 120 kVp and reconstructed with Qr36.

**Results:**

Automatic selection of the optimal tube voltage resulted in a CTDIvol reduction of 22%, 15%, and 12% compared with the reference for the small, medium, and large phantom, respectively. CT numbers differed up to 64% for the standard reconstruction and 11% for the calcium-aware reconstruction. Similarly, Agatston scores deviated up to 40% and 8% for the standard and calcium-aware reconstruction technique, respectively.

**Conclusion:**

CT numbers remained consistent with comparable calcium scores when the calcium-aware image reconstruction technique was applied with varying tube voltage. Less consistency was observed in small calcifications with low density. Automatic reduction of tube voltage resulted in a dose reduction of up to 22%.

**Key Points:**

*• The calcium-aware image reconstruction technique allows for consistent CT numbers when varying the tube voltage.*

*• Automatic reduction of tube voltage results in a reduced radiation exposure of up to 22%.*

*• This study stresses the known limitations of the current Agatston score technique.*

**Electronic supplementary material:**

The online version of this article (10.1007/s00330-020-06709-9) contains supplementary material, which is available to authorized users.

## Introduction

Ischemic heart diseases remain one of the leading causes of death worldwide [1, 2]. Within the framework of individual risk prediction for these diseases, the assessment of coronary artery calcium has become increasingly important. Currently, the most common strategy for quantification of the coronary artery calcium score (CACS) is on computed tomography (CT) examinations using the Agatston method [3]. Despite the excellent prognostic value of this CT-based strategy, the Agatston scoring method has some limitations [4, 5]. Recent guidelines demand a fixed tube voltage of 120 peak kilo voltage (kVp) in combination with filtered back projection (FBP) or iterative reconstruction with 100 kVp acquisition after site- and literature-based validation [5, 6]. However, there is a main argument for the use of lower, or even patient-specific, tube voltages: the need to reduce radiation dose given the increase in the number of CT examinations [7].

Lowering tube voltage potentially reduces radiation dose in CACS at the cost of inconsistent scores because CT numbers, expressed in Hounsfield units (HU), are energy dependent. In this case, the standard calcium scoring threshold should be made tube voltage or patient-specific.

Recently, a calcium-aware reconstruction technique was introduced via the application of a new reconstruction kernel (Sa36f). The technique is also known by the name “Agatston score equivalent calcium scoring,” “artificial 120 kV equivalent CT images,” or “artificial 120.” Please refer to the vendor’s whitepaper for a detailed explanation [8]. With this technique, CT numbers of calcium are scaled to match the CT numbers that would have been measured at 120 kVp, enabling the use of the standard 130 HU threshold [9]. The technique might enable acquiring images at reduced radiation dose, while preserving the Agatston score and its risk assessment potential. In contrast to tube voltage–dependent threshold adjustments, the calcium-aware reconstruction technique seems an easy tool to implement clinically.

The purpose of our phantom study was to evaluate the calcium-aware reconstruction technique with regard to coronary calcium quantification for a wide range of tube voltages and calcifications varying in size and density and for different chest sizes. Moreover, the radiation dose reduction by automatic tube voltage selection was assessed for these cases.

## Materials and methods

### Phantom

An anthropomorphic (cardio) thoracic CT phantom (QRM Thorax, QRM GmbH) in combination with two different inserts was used for quantitative assessment of CACS both for the standard and the calcium-aware reconstruction technique. One insert (D100, QRM GmbH) contained 100 calcifications of different diameters (0.5 to 2.0 mm) and hydroxyapatite (HA) densities (90 to 540 mg HA/cm^3^) [10]. The other insert was a cylindrical cardiac calcification insert (CCI, QRM GmbH) with nine calcifications varying in size (1.0 to 5.0 mm) and density (200 to 800 mg HA/cm^3^). To simulate different chest sizes, the thorax phantom was scanned with and without fat-equivalent extension rings (QRM GmbH) resulting in three different chest sizes: small (300 × 200 mm), medium (350 × 250 mm), and large (400 × 300 mm). To ensure a realistic translation of the results from different phantom sizes to human chest sizes, the water equivalent diameter (Dw) was used. Dw reflects the x-ray attenuation of the patient and is therefore a preferred patient size metric [11]. Retrospective analysis of Dw’s in 41 patient scans for CACS performed in our hospital showed that these diameters mostly matched with the Dw of the medium and large extension rings.

### Acquisition and reconstruction parameters

Scans were performed on a dual source CT (DSCT) system (SOMATOM Force, Siemens Healthineers, Syngo CT VB10). A reference tube voltage of 120 kVp in combination with automated tube current modulation (ATCM) CARE Dose4D was used for both inserts (Table 1). The calcium-aware reconstruction technique was assessed by acquiring data with varying tube voltages of 70–150 kVp, in steps of 10 kVp. Additionally, automatic tube voltage selection (“kVon”) was set to keep the contrast to noise ratio for calcium constant when selecting the optimal tube voltage for radiation dose optimization. Finally, a scan was performed using a dedicated CACS Tin filtration protocol with an adaptation of the reference tube voltage to Sn100 in combination with ATCM CARE Dose4D (Table 1). All scans were repeated five times after manual repositioning (approximately 2 mm translation and 2 degrees rotation) of the phantom to assess positioning influence and interscan variation.

**Table 1:** Acquisition and reconstruction parameters

Scanner*	SOMATOM Force	SOMATOM Force-tin filtration
Acquisition mode	Sequential	Sequential
Scan length (mm)	100.5	100.5
Reference tube voltage	120	Sn100
Reference tube current product	80	534
Manual tube voltage settings	70–150	Sn100
CARE kV dose optimization slider**	5 (bone/calcium)	5 (bone/calcium)
Collimation (mm)	32 × 1.2	32 × 1.2
Rotation time (sec)	0.25	0.25
Image reconstruction (FBP)	Qr36 and Sa36	Qr36 and Sa36
Slice thickness (mm)	3.0	3.0
Increment (mm)***	1.5	1.5
FoV (mm)	180	180
Reconstruction matrix	512 × 512	512 × 512

*Siemens Healthineers, Syngo CT VB10

**The dose optimization slider from the default calcium scoring protocol was retained

***Increment of 1.5 mm is the standard for calcium scoring with Siemens equipment

Images were reconstructed with the conventional calcium scoring reconstruction technique (kernel Qr36) and the dedicated calcium-aware reconstruction technique (kernel Sa36), both based on FBP. For the latter technique, calcium is identified in preliminary reconstructed images and a lookup table is used to correct the CT numbers of calcium in the finally reconstructed images [8]. The exact working of the algorithm is proprietary information of the vendor. The algorithm is fully integrated within the standard image reconstruction interface and can be activated by selecting the corresponding reconstruction kernel (Sa36). It does not need an additional workstation or increased reconstruction times.

### Image and dose analysis

The volumetric CT dose index values (CTDIvol) in mGy were noted to assess potential radiation dose reduction. Consistency of CT numbers (mean and standard deviation (SD)) was determined in the central calcium insert (200HA) of the CCI insert. Noise SD was determined within a homogeneous region of the CCI insert. Agatston score, together with different image quality metrics, was computed using an in-house developed Python script (Python version 3.7) for the D100 and CCI insert. Resulting Agatston scores of the Python script were validated against the standard vendor-specific scoring software (Syngo.via, Siemens Healthineers) with the aid of CCI data and proven equal (maximum deviation 0.1%).

This study addresses directly the CT number or CT value in Hounsfield units (HU) of calcifications. CT numbers are related to the linear x-ray attenuation coefficients and depend on the density, the effective atomic number, and x-ray tube voltage [12]. The attenuation coefficient of the phantom base material does not resemble the attenuation coefficient of human soft tissue equally well at all tube voltages. Allmendinger et al previously described a base material-specific correction, necessary for correct Agatston scores at varying tube voltages by adjustment of the standard 130 HU threshold [8]. This correction was applied automatically in our study as well as for all reconstructions.

Image noise was compared with recommended noise targets (in HU) for calcium scoring CT scans defined for different chest sizes (small, medium, large chest width): 20 HU for the small and medium chest width, and 23 HU for the large chest width [13].

Additionally, an Agatston score was determined in a non-calcium region (55 × 55 mm), therefore depending purely on noise. This score was called the background Agatston score (BAS). For acquisitions with a non-zero BAS, the Agatston scores of calcifications could be less reliable, as it was uncertain if a calcification was seen at a specific location, or just noise. These scores were noted.

Reference values for both inserts were the Agatston scores acquired with a tube voltage of 120 kVp and reconstructed with the standard technique (Qr36). Each deviation in acquisition or reconstruction was compared against this reference

### Statistical analyses

SPSS (version 25, IBM Corp) was used for statistical analysis. Normality of data was tested with the Shapiro-Wilk test. Wilcoxon signed-rank test was performed to evaluate statistically significant difference of the median Agatston scores. Intraclass correlation coefficients (ICC) with a 95% confidence interval (CI) and Bland-Altman plots of the Agatston scores between two different techniques were assessed. A *p* value of < 0.05 was considered statistically significant. Agatston scores are given as median values of the five measurements.

## Results

### Radiation dose and noise values

Reference dose levels at 120 kVp for the small, medium, and large phantom size were 1.57, 2.59, and 3.84 mGy respectively. For the scans with automatic tube voltage selection, tube voltage was reduced to 90 kVp for the small and medium phantom size, while 100 kVp was selected for the large phantom. In comparison with the corresponding reference, radiation dose levels decreased by 22%, 15%, and 12% for the small, medium, and large phantom size, respectively.

Within the dedicated Tin CACS protocol, dose values were 55% lower for both small and medium phantom size and 60% for the large phantom size compared with the reference dose levels at 120 kVp.

**Fig. 1:**
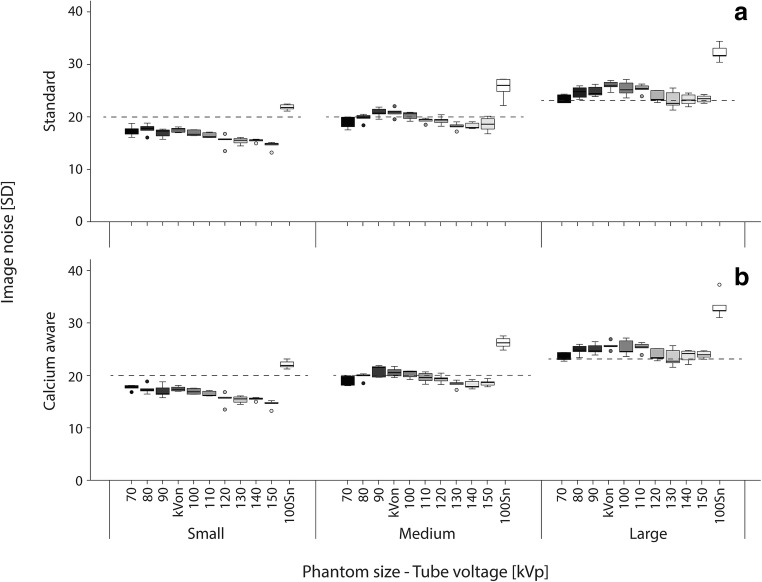
Box-and-whisker plots of the noise measurements of the homogeneous central slice of the CCI insert. Recommended noise targets (in HU) for calcium scoring CT scans defined for different chest sizes were applied to the images as dotted lines: 20 HU for the small and medium chest width, and 23 HU for the large chest width. The automatic tube voltage selection is illustrated by “kVon”

Median noise values for the 120 kVp and the images obtained with automatic tube voltage selection increased with increasing phantom diameter for both reconstruction techniques (Fig. 1). The noise level in all three phantom sizes was highest when using Tin filtration. Moreover, the recommended noise target for calcium scoring CT scans was exceeded for some tube voltages in the medium phantom and for all tube voltages in the large phantom size (Fig. 1). Despite the high number of noise limit exceeding scans, BAS values were zero for most reconstructions. A BAS > 0 was found only for the large phantom in combination with a tube voltage of 70 kVp or Sn100.

### CT number constancy

Considering the large calcification with 200 mg HA/cm^3^ in the CCI insert for all phantom sizes, CT numbers increased with decreasing tube voltage for the standard reconstruction technique, while these numbers remained virtually constant for the calcium-aware reconstruction technique (Table 2). Median HU (min HU–max HU) of the reference (120 kVp + Qr36) was 266 HU (265HU—268HU), 257 HU (257HU—258HU), and 247 HU (246HU—248HU) for the small, medium, and large phantom size, respectively. Compared with the reference, the deviation was up to 64% with the standard reconstruction technique and up to 11% with the calcium-aware reconstruction technique when varying the tube voltage (Table 2).

**Table 2:** Deviation of the CT number of calcium at varying tube voltage and phantom size compared with the reference with a tube voltage of 120 kV and the standard reconstruction technique (Qr36)

	Deviation of the CT number of calcium					
	Calcium-aware reconstruction technique			Standard reconstruction technique		
Tube voltage	Small phantom	Medium phantom	Large phantom	Small phantom	Medium phantom	Large phantom
70	− 11.0% (− 9.9 to − 11.1%]	− 4.3% (− 4.0 to − 5.0%)	2.2% (1.8 to 3.5%)	60.5% (59.6 to 61.6%)	61.0% (60.4 to 62.0%)	63.6% (62.0 to 65.6%)
80	− 7.0% (− 6.7 to − 7.6%)	− 2.7% (− 2.3 to − 2.8%)	3.2% (2.6 to 3.8%)	40.1% (39.4 to 40.4%)	39.1% (39.0 to 40.1%)	40.8% (40.3 to 41.6%)
90	− 3.6% (− 3.4 to − 4.6%)	− 0.5% (− 0.9 to 0.7%)	3.7% (3.6 to 5.4%)	25.4% (25.3 to 26.3%)	24.4% (24.1 to 25.9%)	25.3% (24.2 to 26.5%)
100	− 2.4% (− 2.0 to − 2.9%)	0.6% (0.3 to 1.7%)	4.7% (3.3 to 5.6%)	14.9% (14.6 to 15.3%)	13.6% (13.3 to 15.0%)	14.0% (12.5 to 15.9%)
110	− 2.2% (− 1.7 to − 2.7%)	1.1% (0.8 to 1.3%)	5.2% (4.2 to 5.6%)	6.3% (5.7 to 6.9%)	5.8% (5.6 to 6.2%)	6.4% (5.4 to 6.7%)
120	− 1.5% (− 0.7 to − 2.0%)	2.1% (1.9 to 2.3%)	5.4% (5.1 to 6.0%)	0.0% (− 0.5 to 0.8%)	0.0% (− 0.2 to 0.2%)	0.0% (− 0.3 to 0.6%)
130	− 1.4% (− 0.6 to − 2.3%)	1.9% (0.9 to 2.1%)	5.8% (5.5 to 6.5%)	− 5.3% (− 4.5% to − 6.2%]	− 5.6% (− 5.4 to − 6.3%)	− 4.6% (− 4.0 to − 4.7%)
140	− 0.9% (− 0.8 to − 1.7%)	2.0% (0.8 to 2.9%)	4.7% (4.4 to 6.1%)	− 9.4% (− 9.3 to 10.1%)	− 9.6% (− 8.8 to 10.7%)	− 9.6% (− 8.6 to 10.0%)
150	− 1.1% (− 0.6 to − 1.8%)	1.9% (1.2 to 3.0%)	5.7% (5.2 to 6.5%)	− 13.0% (− 12.6 to − 13.6%)	− 13.2% (− 12.4 to − 13.8%)	− 12.3% (− 11.3 to − 12.5%)
Sn100	− 5.3% (− 4.3 to − 7.0%)	− 2.5% (− 1.4 to − 3.8%)	1.5% (0.2 to 3.7%)	− 16.5% (− 16.2 to − 17.9%)	− 13.9% (− 13.0 to − 14.8%)	− 11.1% (− 9.0 to − 11.8%)

Values given in median% (min%–max%)

### Agatston score

**Fig. 2:**
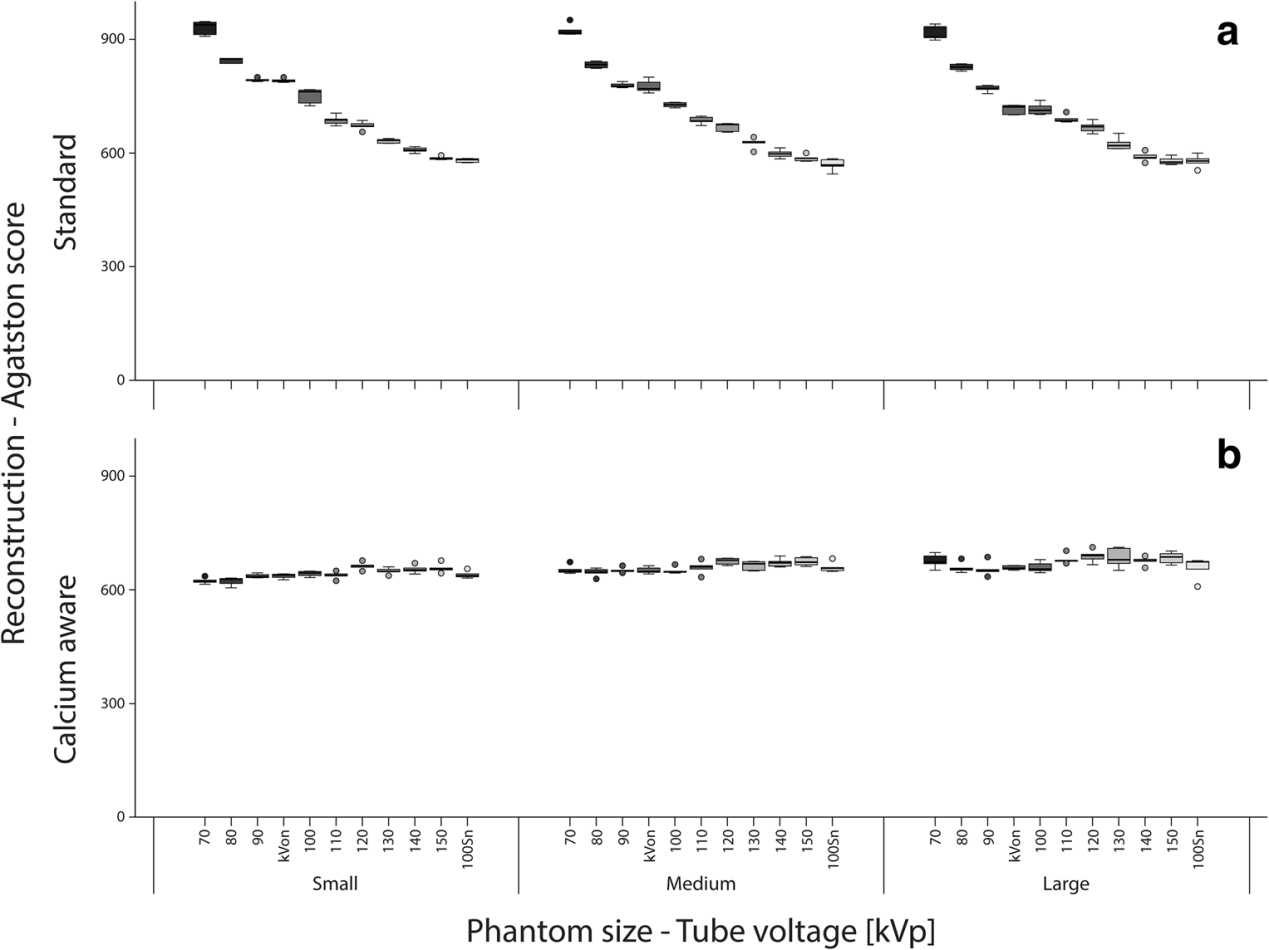
**a** Box-and whisker plots of the Agatston score within the CCI insert with the standard reconstruction technique. **b** Box-and-whisker plots of the Agatston score within the CCI insert with the calcium-aware reconstruction technique. Scores are given per phantom size-tube voltage combination. The automatic tube voltage selection is illustrated by “kVon”

**Fig. 3:**
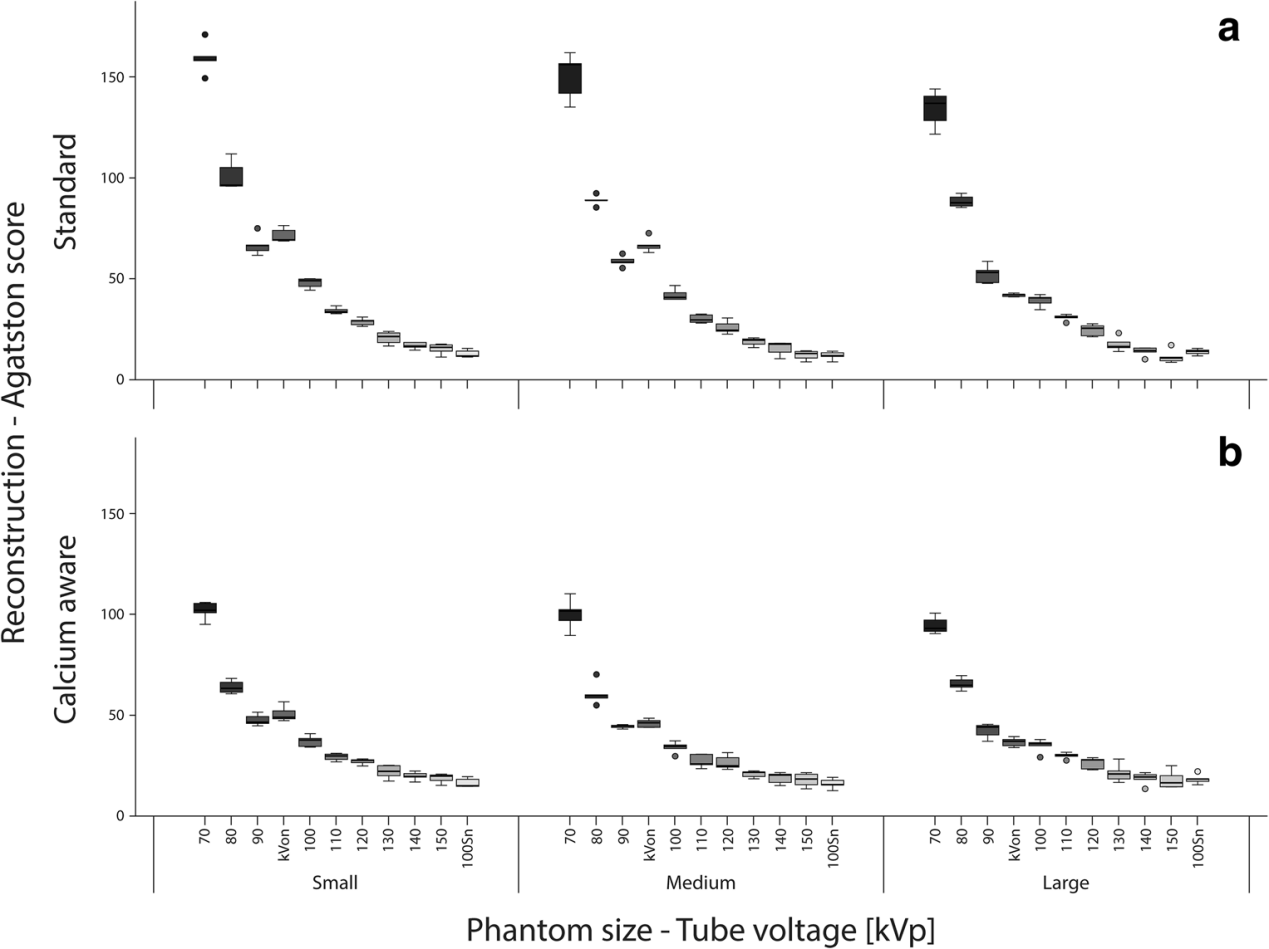
**a** Box-and-whisker plots of the Agatston score within the D100 insert with the standard reconstruction technique. **b** Box-and whisker-plots of the Agatston score within the D100 insert with the calcium-aware reconstruction technique. Scores are given per phantom size-tube voltage combination. The automatic tube voltage selection is illustrated by “kVon”

**Fig. 4:**
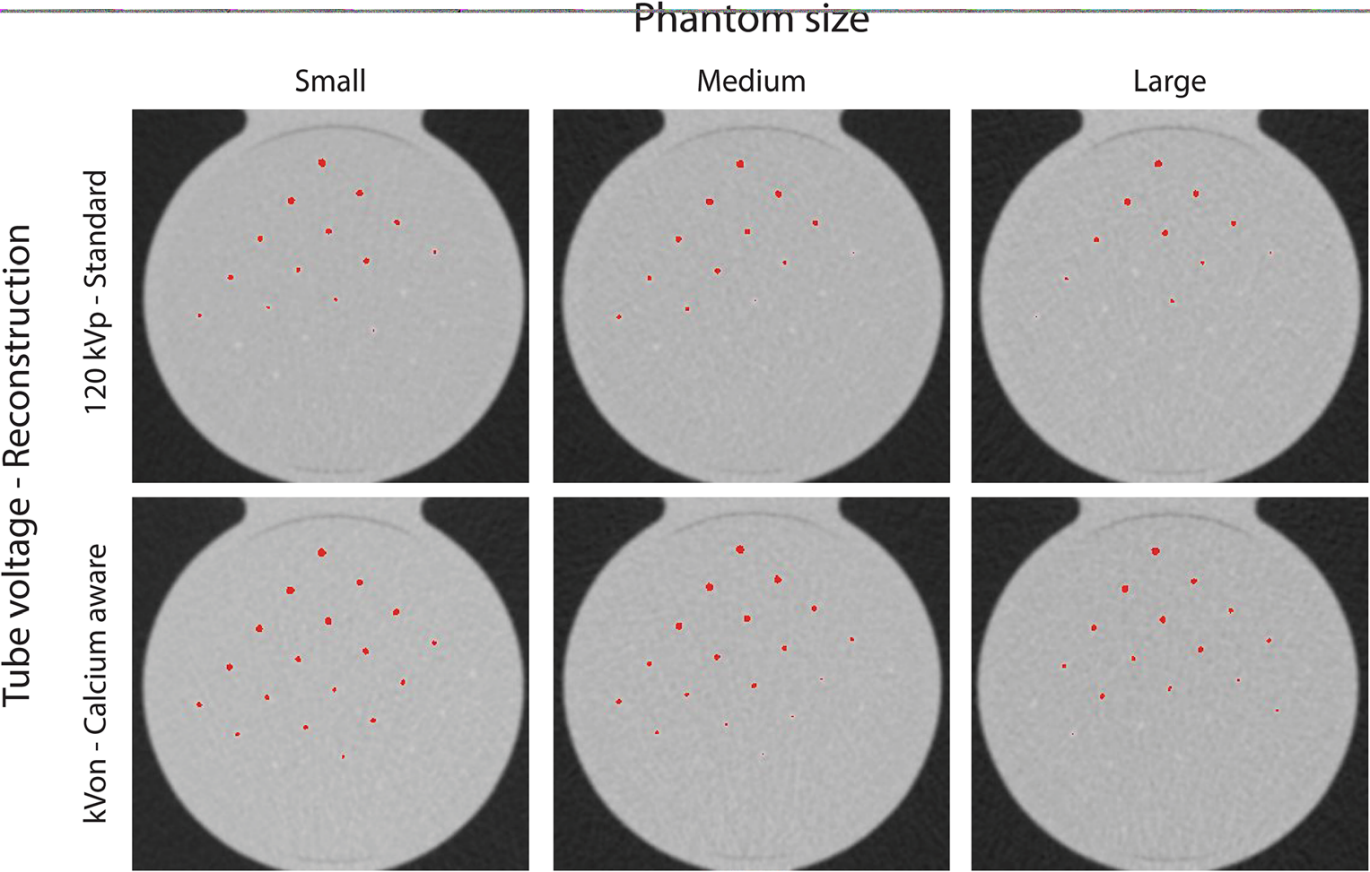
Visualization of calcifications in the D100 insert with all voxels with a CT number above the threshold colored red. From left to right, the phantom size increases. The upper row images were reconstructed with the standard reconstruction technique with a tube voltage of 120 kVp. Lower row images were reconstructed with the calcium-aware reconstruction technique and automated tube voltage selection (90 kVp for the small and medium size phantom and 100 kVp for the large size phantom)

When varying the tube voltage, Agatston scores deviated up to 40% and 8% from the reference for the standard and calcium-aware reconstruction technique, respectively (Table 3). The overall spread in median Agatston scores for varying tube voltages decreased for the calcium-aware reconstruction technique for both the CCI and D100 insert (Fig. 2 and Fig. 3). Considering all phantom sizes, the Agatston scores in the CCI insert increased with 14% for the automated tube voltage selection and decreased with 14% within the tin-filtrated scans for the standard reconstruction technique (Fig. 2a). For the calcium-aware reconstruction technique, Agatston score deviations from the reference were much less: 3.6% at automated tube voltage selection and 2.4% with the tin-filtrated scans (Fig. 2b). For the D100 insert, we observed similar results; however, the deviations from the reference were larger than in the CCI insert, especially for the varying tube voltage in combination with the standard reconstruction technique (Fig. 3). Representative images of the D100 insert for the standard reconstruction technique with 120 kVp and the calcium-aware reconstruction technique at reduced tube voltage for all three phantom sizes are shown in Fig. 4. This figure shows calcifications with an Agatston score of zero for the reference, while the calcium-aware reconstruction technique Agatston scores are non-zero.

**Table 3:** Agatston score deviation at varying tube voltage and phantom size compared with the reference with a tube voltage of 120 kV and the standard reconstruction kernel (Qr36)

	Agatston score deviation					
	Calcium-aware reconstruction technique			Standard reconstruction technique		
Tube voltage	Small phantom	Medium phantom	Large phantom	Small phantom	Medium phantom	Large phantom
70	− 7.5% (− 1.9 to − 10.4%)	− 2.5% (2.6 to − 7.1%)	1.8% (− 3.1 to 5.6%)	39.7% (33.6 to 44.0%)	38.1% (33.0 to 44.3%)	36.7% (31.6 to 43.3%)
80	− 7.0% (− 3.0 to − 11.7%)	− 3.8% (1.7 to − 8.0%)	− 1.6% (5.2 to − 7.0%)	26.3% (21.9 to 28.8%)	24.6% (20.9 to 28.4%)	24.4% (19.1 to 27.7%)
90	− 5.2% (− 1.2 to − 8.8%)	− 2.0% (2.5 to − 7.4%)	− 2.2% (4.3 to − 6.1%)	18.1% (14.2 to 22.3%)	16.8% (11.6 to 20.0%)	14.5% (9.9 to 20.4%)
100	− 3.4% (− 1.2 to − 7.2%)	− 2.8% (1.9 to − 6.8%)	− 1.5% (4.4 to − 5.7%)	13.1% (5.8 to 16.5%)	7.0% (2.7 to 15.8%)	7.7% (1.1 to 13.1%)
110	− 5.6% (− 0.5 to − 8.5%)	− 0.8% (3.2 to − 6.2%)	2.5% (− 3.4 to 6.8%)	1.7% (− 1.3 to 7.4%)	2.2% (− 0.6 to 7.2%)	3.7% (− 2.5 to 8.4%)
120	− 1.1% (3.8 to − 5.6)	0.7% (− 2.8 to 4.6%)	2.2% (− 2.3 to 10.3%)	0.0% (− 4.4 to 5.3%)	0.0% (− 4.6 to 3.9%)	0.0% (− 4.7 to 5.6%)
130	− 3.5% (1.1 to − 5.7%)	− 0.9% (4.9 to − 5.5%)	2.4% (− 5.2 to 10.7%)	− 6.2% (− 2.5 to − 7.5%)	− 6.2% (− 2.0 to − 11.1%)	− 7.5% (− 0.6% to − 11.3%)
140	− 2.6% (2.4 to − 5.8%)	− 0.1% (− 4.1 to 6.8%)	0.6% (− 3.5 to 7.9%)	− 10.2% (− 5.3 to 12.2%)	− 10.5% (− 6.6 to − 14.4%)	− 11.3% (− 6.8 to − 16.5%)
150	− 3.1% (3.1 to − 5.5%)	0.1% (− 2.9% to 6.7%)	1.7% (− 3.4 to 10.1%)	− 12.7% (− 10.0 to − 14.1%)	− 11.3% (− 8.8 to − 16.8%)	− 14.5% (− 7.2 to − 17.1%)
Sn100	− 4.4% (0.1 to − 8.7%)	− 2.5% (5.6 to − 7.8%)	− 0.4% (9.3 to − 17.4%)	− 13.7% (− 9.7 to − 17.2%)	− 15.7% (10.5 to − 41.0%)	− 12.2% (− 3.0 to − 28.3%)

Values given in median% (min%–max%)

**Fig. 5:**
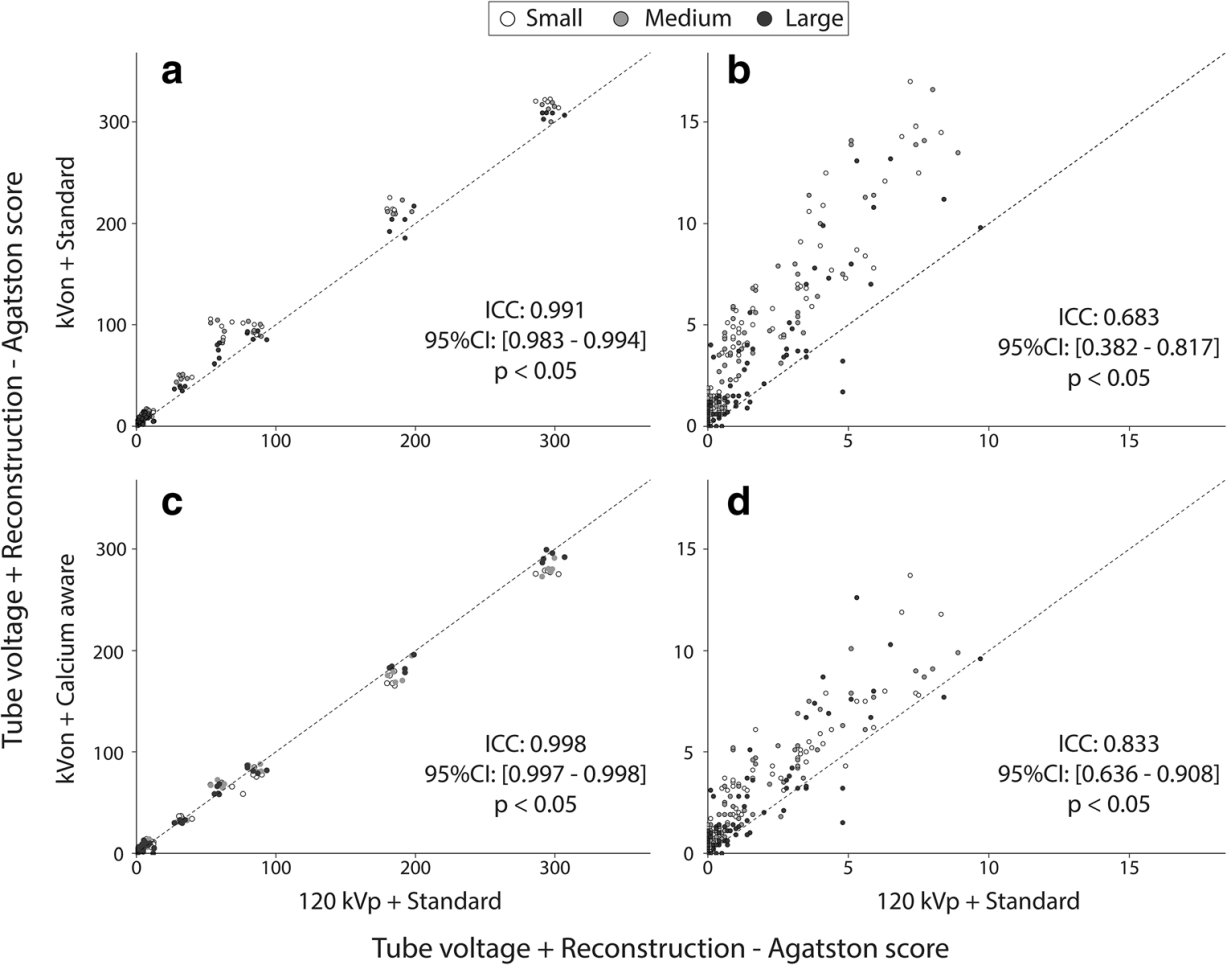
The ICC of the Agatston score for the small, medium, and large phantom for. **a** The standard reconstruction technique with automatic tube voltage selection compared with the standard reconstruction with 120 kVp. **b** Detail of the graph in **a** representing the low density and small calcifications. **c** The calcium-aware reconstruction technique with automatic tube voltage selection and the standard reconstruction with 120 kVp. **d** Detail of the graph in **c** representing the low density and small calcifications

**Fig. 6:**
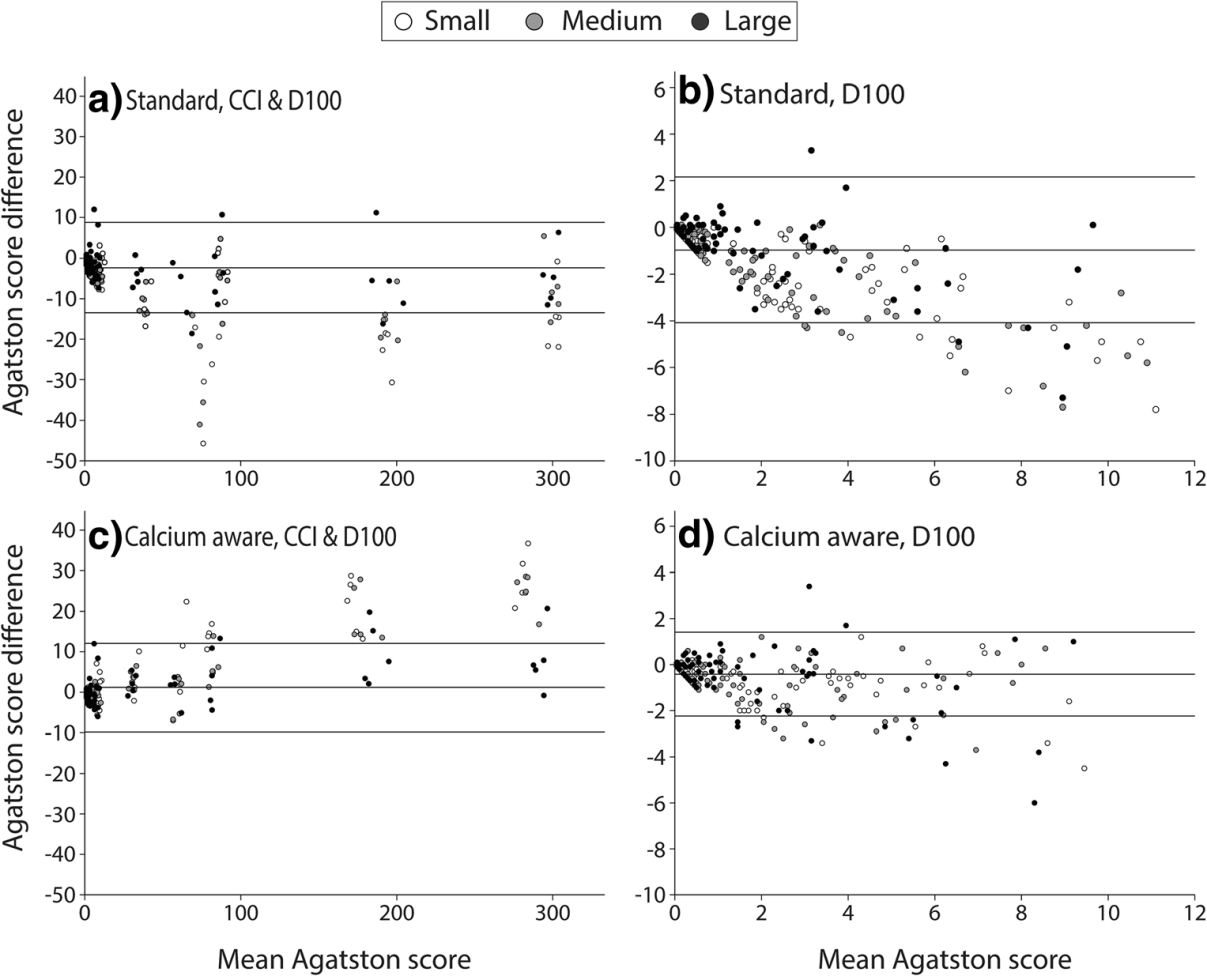
Bland-Altman Plots with mean difference and 95% limits of agreement for the small, medium, and large phantom with the CCI and/or D100 insert. All plots show an Agatston score comparison between the reference at 120 kVp (with standard reconstruction technique) and scans with automatic tube voltage selection (with standard reconstruction technique (**a**, **b**) and with calcium-aware reconstruction technique (**c**, **d**))

There was a very high ICC (0.991) and 95% CI for the automated tube voltage selection with the standard reconstruction technique compared with the reference when considering all calcifications (Fig. 5a). When considering only the low Agatston scores, both the ICC and 95% CI decreased (Fig. 5b). There was a very high ICC (0.998) and 95% CI for the automated tube voltage selection and the calcium-aware reconstruction technique compared with the reference (Fig. 5c). When considering only the low Agatston scores, both the ICC and 95% CI decreased (Fig. 5d). However, this decrease was less than observed within the standard reconstruction technique. A Bland-Altman analysis of the data is shown in Fig. 6. The Bland-Altman plots demonstrate the agreement between the two reconstruction kernels. The negative mean difference within Fig. 6 a, b, and d demonstrates that, regardless of reconstruction technique, Agatston scores are higher for automatic tube voltage selection in comparison with 120 kVp. The opposite applies for the calcium-aware reconstruction technique and automatic tube voltage selection (Fig. 6c).

For the CCI insert, increasing the phantom diameter from small to large demonstrated no statistically significant decrease (*p* = 0.5) of the median (range) Agatston scores from 671 (656.2—686.5) to 669.9 (651.1—689.4) for the reference (120 kVp and Qr36). A statistically significant increase (*p* < 0.05) in Agatston score from 639 (626.9—642.4) to 657.4 (652–664.5) was observed for the calcium-aware technique with automated tube voltage selection. For the D100 insert and increasing phantom size from small to large, there was a statistically significant decrease (*p* < 0.05) in Agatston score from 29.3 (26.5—31.2) to 25.6 (21.4—27.8) for the reference (*p* < 0.05), and a statistically significant decrease (*p* < 0.05) in Agatston score from 49.0 (47.4—56.7) to 37.1 (34.0—39.4) for the calcium-aware reconstruction technique with automatic tube voltage selection.

## Discussion

Our results demonstrate that CACS with a calcium-aware image reconstruction technique allows for consistent CT numbers when varying the tube voltage and allows for reduced radiation exposure with automatic reduction of tube voltage.

The Agatston scores with the calcium-aware reconstruction technique deviated up to 8% for the calcifications of the CCI insert across 70 to 150 kVp and Sn100, whereas the Agatston score with the standard reconstruction deviated much more with up to 40%. The latter might be explained by the increase of the photo-electric effect for calcium when scanning with low tube voltage settings. In contrast to the CCI insert, Agatston scores were not stable for the calcifications of the D100 insert when varying the tube voltage. For the calcium-aware reconstruction technique, this might be explained by a sub-optimal identification of the voxels containing calcifications of small diameter and low density and subsequently a sub-optimal correction of the CT numbers.

As seen in Fig. 4, there were additional calcifications detected when lowering tube voltage. Thus, it might be possible that a patient with a zero Agatston score at 120 kVp might have a non-zero Agatston score at a lower tube voltage, despite the application of the calcium-aware reconstruction technique. This might influence the work-up of patients suspected for coronary artery disease. However, the increase of Agatston score in the D100 insert, as demonstrated in Fig. 4, is due to true calcified lesions. Instead of improving the calcium-aware reconstruction technique presented in this study to better resemble the Agatston scores at 120 kV, we prefer to reinvent calcium imaging and think it is time to let go the conventional scoring method [14, 15]. For example, Groen et al described a correction applied to the 130 HU calcium scoring threshold for the increased CT numbers of calcium when varying tube voltage and applying the standard reconstruction technique [16].

Our study demonstrated a decrease in Agatston score with increasing phantom size, as previously described for the standard reconstruction technique and the D100 insert [17]. However, our study used both the CCI and the D100 insert and in addition the calcium-aware reconstruction technique. We observed an increase of the Agatston score for the CCI insert when using the calcium-aware reconstruction technique. The increase in Agatston score might be explained by the sub-optimal identification of the voxels containing small and low-density calcifications, while noise increased.

Calcium CT numbers were constant for the calcium-aware reconstruction technique with automated tube voltage selection, irrespective of phantom size. However, Agatston scores varied more than the reference for different patient sizes. The reason for this is twofold. First, the constancy of CT numbers is calculated as the mean of a large ROI enclosing the calibration rod of the CCI phantom, while Agatston scores are calculated for the smaller nine calcifications. Second, despite the use of clinical scan protocols, higher noise levels were shown especially for the lower tube voltages and the automated tube voltage selection (Fig. 1). Our computation of the Agatston score was validated to the standard vendor-specific software, calculating every single voxel above a threshold of 130 HU for CACS. With higher noise levels, Agatston scores also increase.

Technological developments like tin filtration and automated tube voltage selection allow for a substantial dose reduction. For example, a 100 kVp with tin filtration CACS protocol demonstrated similar Agatston scores as the reference protocol with 120 kVp despite using the standard reconstruction technique [18]. Larger deviations are expected for tube voltages like 70 and 80 kVp (Table 2). A great advantage of the currently considered calcium-aware reconstruction technique is that CACS can be obtained more accurately from any acquisition, regardless of applied tube voltage and filtration. This allows for CACS to be considered within cancer screening protocols. The use of a CACS with the aid of tin filtration combined with an early prototype of a calcium-aware reconstruction technique was described in a patient study and considered to be potentially feasible for calcium scoring [19]. However, in this study and our study, an increased image noise for the tin-filtrated scans was observed. The noise levels were above the recommended noise levels by the SCCT in all three phantom sizes, especially for the large phantom size. Possible solutions for sub-optimal identification of calcification when applying tin filtration with increased noise levels are proposed, e.g., a HU threshold correction for CACS [20] or investigation to apply iterative reconstructions. Within our study, we observed BAS of > 0 for the tin-filtrated vendor-recommended scans in the large phantom size. Therefore, caution must be taken when applying the tin-filtrated scans in clinical routine, especially when CACS is obtained for calcification of small diameter and low density, as the calcium-aware reconstruction technique is also not able to correct these.

The recommended noise levels were not only exceeded for the tin-filtrated scanning protocols, but also for all tube voltage settings within the large phantom diameter, despite the use of the vendor-recommended scanning protocols. This warrants further investigation for adjusting the reference tube current value or the adaptation strength of the CARE Dose4D dose curve to achieve the recommended noise target level [13]. However, it seems that the recommended noise target limit comes with a very safe margin. After all, the BAS was zero for all reconstructions in the small- and medium-sized phantoms and for the calcium-aware reconstruction technique with automated tube voltage selection in all phantoms.

There are limitations in this study that need to be considered. This study was phantom-based and despite the effort to represent clinical routine, patient studies are necessary to validate our findings. CTDIvol is an indicator of the CT scanner radiation output. The dose received by a patient depends on this CTDIvol and the individual patient size. It is recommended to use the size-specific dose estimates (SSDE) to reflect estimated doses for the individual patient [21]. Furthermore, it might be of interest to use a non-stationary phantom model instead of a stationary one. This makes it feasible to assess whether or not heart rate variability will influence Agatston scores when using the calcium-aware reconstruction technique.

## Conclusion

In general, CT numbers remained consistent with comparable calcium scores when the calcium-aware image reconstruction technique was applied with varying tube voltage. Less consistency was observed in small calcifications with low density. Automatic reduction of tube voltage resulted in a dose reduction of up to 22%.

## Electronic supplementary material

ESM 1 (DOCX 6337 kb)

## Abbreviations

ATCM: Automated tube current modulation
BAS: Background Agatston score
CACS: Coronary artery calcium scoring
CI: Confidence interval
CNR: Contrast to noise ratio
CT: Computed tomography
CTDI: Computed tomography dose index
DSCT: Dual source computed tomography
Dw: Water equivalent diameter
FBP: Filtered back projection
FoV: Field of view
HU: Hounsfield unit
ICC: Intraclass correlation coefficient
SD: Standard deviation
