# Supplementary material for: Dose reduction for CT coronary calcium scoring with a calcium-aware image reconstruction technique: a phantom study
Source: Eur Radiol. 2020 Feb 19;30(6):3346–55. doi: 10.1007/s00330-020-06709-9 (PMC7248036; doi:10.1007/s00330-020-06709-9)
Supplement: Supplementary file 1 — (DOCX 6337 kb) [file 330_2020_6709_MOESM1_ESM.docx]

**
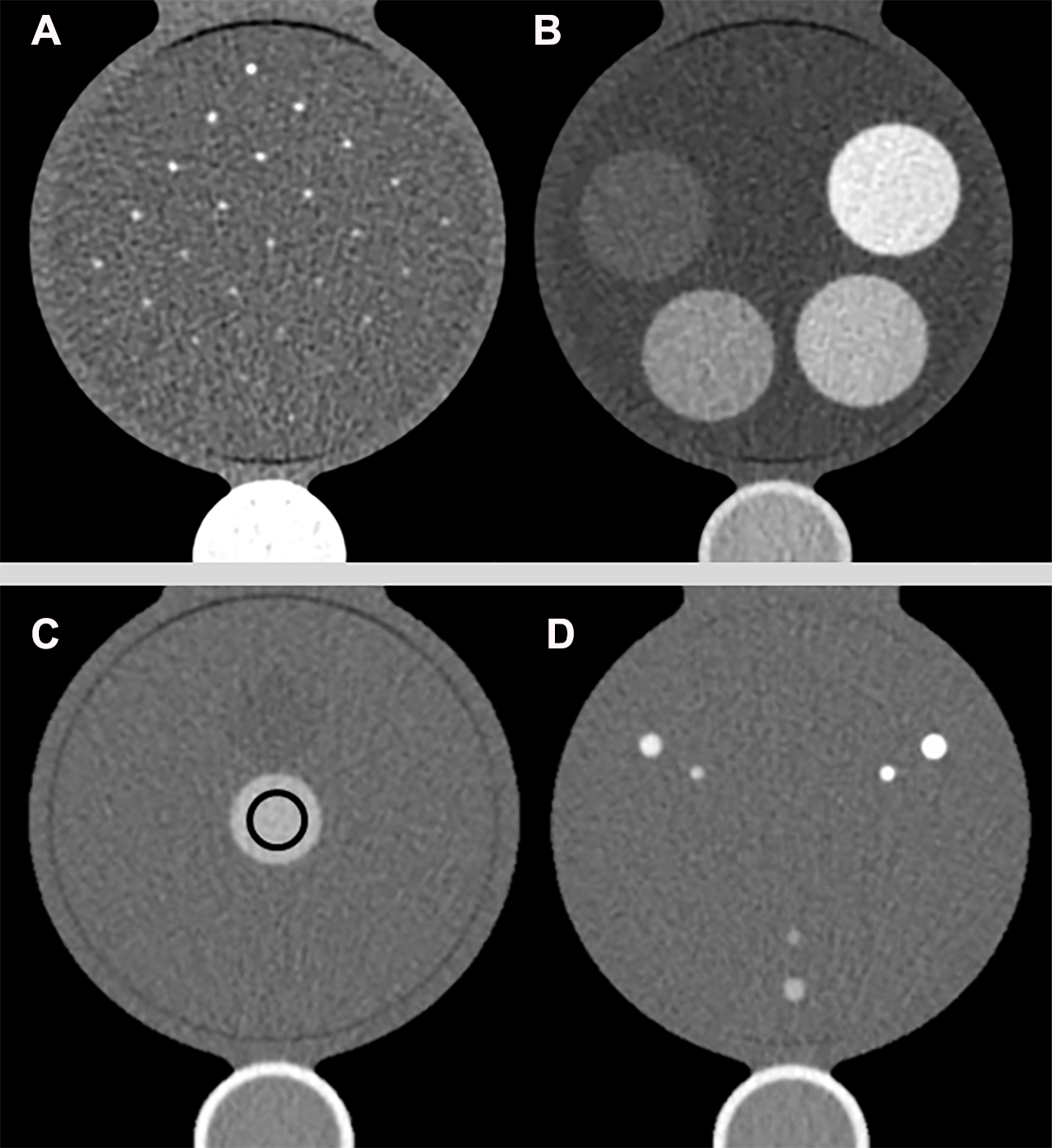
Phantom inserts a** Calcium cylinders of the D100 insert (0.5 mm – 2.0 mm). **b** Calibration rods of the D100 insert (30 mm), nominal hydroxyapatite (HA) 90 – 540 mgHA/cm^3^. **c** HU measurement of the central calcium insert in the CCI insert. **d** Cylindrical calcification inserts of three different HA densities: 800 mgHA/cm^3^ at 2 o'clock position, 200 mgHA/cm^3^ at 6 o'clock position, and 400 mgHA/cm^3^ at 10 o'clock position of the CCI insert.
